# Age‐specific all‐cause mortality rates among adolescents and youth living with and without HIV: Evidence from a cohort study in South Africa

**DOI:** 10.1002/jia2.26522

**Published:** 2025-06-25

**Authors:** Siyanai Zhou, Elona Toska, Bulelani Gwampi, Leigh F. Johnson, Janke Tolmay, Wylene Saal, Zea Leon, Lucia Knight, Lucie Cluver

**Affiliations:** ^1^ Centre for Social Science Research University of Cape Town Cape Town South Africa; ^2^ Division of Social and Behavioural Sciences School of Public Health Faculty of Health Sciences University of Cape Town Cape Town South Africa; ^3^ Department of Sociology University of Cape Town Cape Town South Africa; ^4^ Oxford Research South Africa University of Oxford Oxford UK; ^5^ Centre for Infectious Disease Epidemiology and Research University of Cape Town Cape Town South Africa; ^6^ School of Public Health University of the Western Cape Bellville South Africa; ^7^ Department of Social Policy and Intervention University of Oxford Oxford UK; ^8^ Department of Child and Adolescent Psychiatry University of Cape Town Cape Town South Africa

**Keywords:** all‐cause mortality, adolescents, antiretroviral adherence, antiretroviral treatment, South Africa, vertical transmission

## Abstract

**Introduction:**

Mortality among adolescents living with HIV (ALHIV) remains a global health problem. We lack granular (age‐ and sex‐disaggregated) data on mortality among ALHIV, hence, this study aims to assess all‐cause mortality among ALHIV in a low‐resource setting.

**Methods:**

All adolescents ever initiated on antiretroviral treatment (ART, *N* = 1107) and their HIV‐negative peers (*N* = 456) aged 10–19 years, recruited as part of the Mzantsi Wakho study cohort, were followed up between 2014 and 2022 (yielding 12,427.7 person‐years of follow‐up). First, we assessed the proportion of deaths and estimated crude mortality incidence rates per 100 person‐years of follow‐up and their 95% confidence intervals, stratified by HIV status, sex and mode of HIV acquisition (vertical vs. sexual). We then estimated adjusted incidence rate ratios (IRRs) using Poisson regression adjusted for time‐varying age, sex and time on ART. Last, we used the Cox proportional hazards regression model to estimate the risk of death by ART adherence.

**Results:**

A total of 1563 adolescents and young people were included in this analysis, 70.8% ALHIV and 57% female. More deaths occurred in ALHIV compared to their HIV‐negative peers (8.3% vs. 0.4%, *p*<0.001). Among ALHIV, we observed a significantly higher proportion of deaths among males compared to females (10.7% vs. 7.1%, *p* = 0.036). Overall, mortality increased significantly with age, and males had a higher risk of mortality compared to females. Adolescents and youth living with vertically acquired HIV had a higher risk of mortality than those living with sexually acquired HIV. Comparing mortality rates by mode of HIV acquisition stratified by age and sex, mortality risk was higher among females aged 20+ years with vertically acquired HIV (IRR: 3.61, 95% CI 1.48–8.82) compared to females with sexually acquired HIV of the same age group. In a sub‐sample analysis, sustained ART adherence was associated with a lower risk of death (aHR: 0.44, 95% CI 0.23–0.85).

**Conclusions:**

ALHIV experience higher all‐cause mortality than their HIV‐negative peers, despite having initiated ART. Among ALHIV, mortality risk was higher among males and adolescents who acquired HIV vertically. Strategies to improve survival among ALHIV, including adolescent‐tailored care and support for adherence to ART, are urgently needed.

## 1 INTRODUCTION

Adolescents and youth represent a growing proportion of people living with HIV worldwide [1]. About 90% of the world's population of adolescents living with HIV (ALHIV) live in sub‐Saharan Africa [2, 3, 4] and South Africa has the highest number of ALHIV [5]. In 2023, an estimated 624,567 adolescents and youth, 10–24 years, were living with HIV in South Africa [6, 7]. Despite being initiated on antiretroviral treatment (ART), adolescents continue to experience life‐threatening health vulnerabilities, which significantly impact their survival [1, 8, 9].

Studies of mortality in adults who initiate ART—in early disease—generally show their life expectancies as similar to HIV‐negative adults of the same age [10, 11]. However, few similar investigations exist for ALHIV. Existing evidence shows that mortality among ALHIV remains high compared to other age groups [12, 13]. Suboptimal adherence to ART is one factor that makes adolescents vulnerable to morbidity and mortality [3, 14], and adolescent‐centred interventions can improve health outcomes and reduce mortality in this population group. However, there is a shortage of disaggregated data on mortality for this group to guide these interventions, or to inform the most widely used global estimates of HIV indicators, such as UNAIDS estimates [12]. Current data are characterized by inconsistencies due to small sample sizes, limited follow‐up periods and gaps [15]. Other data from clinics or hospitals do not include comparisons with HIV‐negative peers, and there is limited data on whether adolescents who are “lost‐to‐follow‐up” (LFTU) have dropped out of care, moved to other treatment services or died.

The objectives of this paper are two‐fold. First, we explore incidence rates of all‐cause mortality among adolescents disaggregated by HIV status and sex, and compare age‐adjusted, sex‐stratified mortality rates by mode of HIV acquisition (vertical vs. sexual). Second, we explored differences in all‐cause mortality rates by ART adherence using cohort data from ALHIV.

## 2 METHODS

### 2.1 Study design and setting

This prospective cohort study of ALHIV was conducted in the Eastern Cape province of South Africa between 2014 and 2022. The Eastern Cape is characterized by social and human development challenges, poor infrastructure and poverty [16]. The province has an estimated overall HIV prevalence of 13.7% and an adult prevalence of 18.8% (ages 15–49) as of 2022.

### 2.2 Study population, sampling and data collection

The study's primary focus was on ALHIV, and it followed a four‐step sampling strategy. First, we mapped all ART‐providing health facilities (*N* = 81) in the study area. Second, a health facility was selected if it provided care to at least five ALHIV, was a government facility and had a register with records of all patients, including those LFTU. Fifty‐two health facilities—nine hospitals, five community health centres and 38 primary care clinics—were selected using these criteria. Third, a roster of all eligible adolescent participants from the 52 facilities was prepared, including their clinic records (not older than 3 years). Fourth, all adolescents aged 10–19 years who had ever initiated or been on ART were approached to participate in the study. Study recruitment started at health facilities where adolescents had received or were receiving treatment and care. To ensure a representative sample of ALHIV, participants were traced to their communities, homes or schools, including those who had disengaged from care or were LTFU. To avoid unintended disclosure and prevent potential stigmatization of ALHIV, an additional cohort of same‐age, HIV‐negative peers (*N* = 456) from the neighbouring or same home of the participant were invited to participate in the study. The same consent processes were followed, and this recruitment strategy ensured that participants shared similar environmental and socio‐economic conditions. Initially, recruitment focused on ALHIV; recruitment of HIV‐negative peers was introduced 3 months into the study, leading to a smaller comparison group. In total, 1563 adolescents were followed up three times between 2014 and 2018. At each round, adolescents completed a self‐reported questionnaire on their health experiences at home, in their communities and in healthcare settings, with the support of research assistants trained in working with vulnerable adolescents. An additional follow‐up check‐in was conducted for all participants between 2021 and 2022 using a shortened version of the questionnaire to assess their availability for future follow‐ups, as well as their mobility and mortality status. The time lag between the initial follow‐up and this check‐in was due to funding constraints and logistical challenges related to COVID‐19.

### 2.3 Ethics approval

Ethical approvals were obtained from the University of Cape Town (UCT/CSSR/2013/4, UCT/CSSR/2022/01 and UCT/CSSR/2019/01) and Oxford University (Oxford/CUREC2/12‐21). Data‐sharing agreements were obtained from provincial Departments of Health and Education, and the review boards of participating healthcare facilities. Participants and their caregivers (when adolescents were <18 years) provided voluntary written informed consent for participation at all study waves, including follow‐up check‐ins. All participants received a certificate of participation, snacks and a small gift pack. Adolescents who refused to participate received snacks and a small gift pack.

### 2.4 Outcome: all‐cause mortality

We ascertained all‐cause mortality through community‐based reporting, that is reports from caregivers or relatives of the adolescents followed up between 2014 (baseline) and 2022. Baseline was defined as the date of study entry (Wave 1 interview date). Follow‐up time included the time from the baseline until death (for those who died) or 8 November 2022 (for living participants). Person‐time was defined as an estimate of the actual time‐at‐risk, in years, that all participants contributed to the study (calculated for the follow‐up period: 2014–2022). For this analysis, data for all participants were censored when the following appeared first: (1) death; (2) lost‐to‐study follow‐up (censored at the last interview or contact date); and (3) alive (censored at the end of follow‐up). Since we did not have the exact date of death for 65.3% of participants, we used the median dates between the previous date of contact and the latest date of follow‐up as an estimate of the date of death. The date of death was often missing as it relied on caregiver or relatives’ recall, and, in some cases, was affected by time gaps between death and follow‐up contact.

### 2.5 Key variables

*HIV status*: At baseline, HIV status for all participants was determined through a multi‐step verification process. All potential participants were screened by trained research assistants using a health history approach, which included pre‐agreed questions about whether adolescents had ever been sick or on long‐term medication for ≥2 weeks. HIV‐positive status was then verified through a combination of adolescents’ self‐reports during health screening, caregiver or healthcare provider confirmation and clinic records. Additionally, the HIV status variable was validated through laboratory test data from the National Health Laboratory Service of South Africa in 2022. *Mode of HIV acquisition* (vertical vs. sexual) was determined by following standard methods in sub‐Saharan African paediatric cohorts: age of ART initiation ≤10 years [17, 18]. The age of ART initiation (cut‐off age: before 10 years) was selected as a conservative proxy for participants who acquired HIV through mother‐to‐child transmission. This allocation was validated and updated using a detailed algorithm that considered other factors (i.e. self‐reported sexual history and parental death) in the absence of definitive clinic notes or data ascribing mode of HIV acquisition [19]. *ART adherence*: Five self‐reported measures of adherence were evaluated for sensitivity in detecting elevated viral load [20]. These measures included missed doses in the past 3 days, last missed dose (in the past week and past month) adapted from the Patient Medication Adherence Questionnaire [21] and the clinic appointment measure added based on other study recommendations [22]. All five measures had high test accuracy in detecting elevated viral load (sensitivity over 75%) and were significantly associated with viral load [20]. The five measures were used to categorize adherence over the three time points, into four distinct longitudinal trajectories using group‐based trajectory modelling [23]. The four distinct adherence trajectories: consistent adherence, low start and increasing adherence, gradually decreasing adherence, and low start and decreasing adherence are described in more detail elsewhere [23]. The derived categorical ART adherence trajectories were used as a predictor in the sub‐sample analysis assessing differences in mortality rates by adherence patterns. We defined *sustained adherence* as 1 if the participant was categorized in the consistent adherence trajectory and 0 otherwise.

*Socio‐economic factors* included sex, rural residence, food insecurity, lack of access to basic necessities, double orphanhood (maternally and paternally orphaned) and school non‐enrolment. Lack of access to basic necessities was defined based on lacking access to any of the following eight basic necessities: clothing, a doctor, school fees, shoes, toiletries, uniforms and school equipment. These items were selected as necessities by over 80% of respondents in a nationally representative South African survey [24].

### 2.6 Statistical analysis

Analyses were conducted in Stata (version 17.0, College Station, StataCorp LP, TX). First, we calculated the all‐cause mortality rates stratified by HIV status and sex. We then described mortality outcomes among ALHIV by mode of HIV acquisition. Second, we estimated crude mortality rates per 100 person‐years of follow‐up, stratified by time‐updated age groups (10–14, 15–19 and 20+ years) and sex across adolescent HIV acquisition groups (vertical and sexual). Mortality rates were calculated as the number of deaths divided by the total number of person‐years of follow‐up for each age group and sex stratum using the *stptime* function in Stata. Third, we used Poisson regression with robust variances [25] to estimate adjusted incidence rate ratios (IRRs) of mortality and their 95% CIs for ALHIV with vertically acquired HIV compared to those with sexually acquired HIV. All IRRs were adjusted for time‐varying age (10–14, 15–19 and 20+ years), sex and time on ART. We further fitted a Poisson regression model to estimate overall mortality IRRs and their 95% CIs, controlling for time‐updated age groups, baseline demographic characteristics and socio‐economic factors described above.

Fourth, we used the Kaplan–Meier approach to describe differences in the cumulative incidence of mortality by sustained adherence. The log‐rank test was used to determine the statistical significance of the mortality differences. Fifth, we use the Cox proportional hazards regression model to estimate the risk of all‐cause mortality (death) by ART adherence, adjusting for age, sex, mode of HIV acquisition and time on ART. Lastly, we conducted a sensitivity analysis on the multivariable Cox regression model by changing the date of death to the mid‐point date between the date of last contact and the median follow‐up date for all participants. Since participants enter the study at different ages, suggesting that age is a left‐truncated variable, we utilized a delayed entry approach by setting the study entry age as the time point from which participants were considered at risk for both Kaplan–Meier and Cox regression analysis. This method ensured that participants were not included in the at‐risk set before their study entry age, accounting for the varying ages at study entry. This is a sub‐sample analysis of those who completed the questionnaire at all three time points, with observation time starting after the third follow‐up visit, to avoid immortal time bias [26, 27]. Since age is time‐varying, to estimate mortality by age‐at‐risk, a lexis expansion [25] was conducted to discretize the time‐to‐event variable into 1‐year intervals—structuring person‐time and events into repeated 1‐year intervals per individual—across all time‐to‐event analyses using Stata's *stsplit* command.

## 3 RESULTS

Overall, 1563 adolescents aged 10–19 years at baseline had a follow‐up (post‐ART initiation) between 2014 and 2022, with a loss‐to‐study follow‐up of 7.9% at the latest follow‐up, yielding 12,427.7 person‐years of follow‐up. Table 1 shows the characteristics at baseline and mortality outcomes by HIV status. LTFU did not significantly differ between ALHIV and HIV‐negative peers, by mode of HIV acquisition (vertical vs. sexual transmission) or by other baseline socio‐economic factors, but it was significantly higher among males (18% vs. 0.3%) than females. The majority of the participants were living with HIV and contributed 8722.7 person‐years of follow‐up, while HIV‐negative participants contributed 3705 person‐years of follow‐up. Across these two groups, the proportion of female participants was similar (57% for ALHIV vs. 60.3% for HIV‐negative peers). Mortality was high among ALHIV, and they were 18 times more likely to die during adolescence than their HIV‐negative peers: (8.7% vs. 0.4%, *p*<0.001) over the 8 years. Overall, the proportion of deaths was significantly higher among ALHIV compared to HIV‐negative peers across all measured socio‐economic factors: sex, rural residence, food insecurity, lack of access to basic necessities, double orphanhood and school non‐enrolment. Among ALHIV, we observed a significantly higher proportion of deaths among males compared to females (10.7% vs. 7.1%, *p* = 0.036).

**Table 1:** Baseline characteristics and mortality among adolescents and youth in a South African cohort (*N* = 1563) by HIV status

	ALHIV	Adolescents not living with HIV
**Overall (*n*, %)**	1107 (70.8%)	456 (29.2%)
**Person‐years**	8722.7	3705
**Baseline characteristics**		
Age (years): median (Interquartile range)	13 (11−16)	15 (12−17)
Female (*n*, %)	631 (57%)	275 (60.3%)
**Reported deaths**		
Overall (*n*, %)	96 (8.7%)	2 (0.4%)
Sex (*n*, %)		
Male	51 (10.7%)	1 (0.6%)
Female	45 (7.1%)	1 (0.4%)
Rural residence	20 (7.1%)	2 (1.6%)
Food insecurity	20 (10.6%)	1 (1.3%)
Lack of access to basic necessities	68 (9.4%)	2 (0.7%)
Double orphanhood	17 (10.4%)	0 (0%)
Not enrolled in school	7 (10.3%)	0 (0%)

### 3.1 Mortality outcomes among ALHIV

Table 2 shows the characteristics of mortality for ALHIV. Among ALHIV, 833 (75.2%) acquired HIV vertically and contributed 6625.2 person‐years with a median age at ART initiation of 6 years, and a median duration on ART at baseline of 6 years. The remaining 274 acquired HIV sexually and contributed 2097.5 person‐years with a median age at ART initiation of 16 years and a median time on treatment of 1 year. Overall, females comprised a higher proportion of sexually acquired HIV than vertically acquired HIV (75.9% vs. 50.8%, *p*<0.001). Overall, the rates of mortality for both adolescent HIV acquisition groups were comparable (8.9% vs. 8.0%). Comparing rates by sex, we observed a higher proportion of deaths among males than females for both those who acquired HIV vertically (10.5% vs. 7.3%) and sexually (12.1% vs. 6.7%). The proportion of deaths was similar across both groups, including by sex, rural residence, orphanhood and lack of access to basic necessities. Food insecurity was slightly higher in the sexually acquired group (15.3% vs. 8.5%), while not being in school was highest in those with vertically acquired HIV (14.3%).

**Table 2:** Baseline characteristics and mortality among adolescents and youth in a South African cohort (*N* = 1107) by mode of HIV acquisition

	Adolescents living with vertically acquired HIV	Adolescents living with sexually acquired HIV
**Overall (*n*, %)**	833 (75.2%)	274 (24.8%)
**Person‐years**	6625.2	2097.5
**Baseline characteristics**		
Female (*n*, %)	423 (50.8%)	208 (75.9%)
Age at ART initiation (years): median (Interquartile range)	6 (2−9)	16 (12−18)
Time on ART: median (Interquartile range)	6 (3, 10)	1 (1, 3)
**Reported deaths**		
Overall (*n*, %)	74 (8.9%)	22 (8.0%)
Sex (*n*, %)		
Male	43 (10.5%)	8 (12.1%)
Female	31 (7.3%)	14 (6.7%)
Rural residence	15 (7.3%)	5 (6.8%)
Food insecurity	11 (8.5%)	9 (15.3%)
Lack of access to basic necessities	51 (9.4%)	17 (9.4%)
Double orphanhood	13 (10.1%)	4 (11.4%)
Not enrolled in school	2 (14.3%)	5 (8.6%)

**Figure 1:**
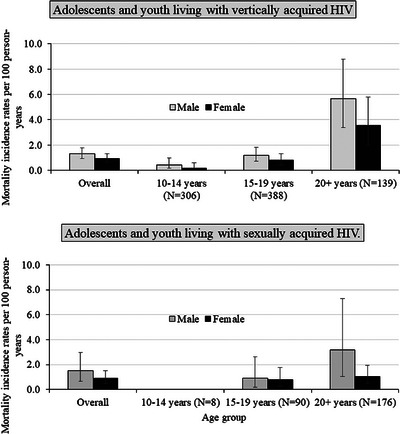
Mortality incidence rates per 100 person‐years and 95% confidence intervals, by time‐updated age and sex among ALHIV.

Among adolescents and youth living with vertically acquired HIV, the overall mortality incidence rate was 1.12/100 person‐years (95% CI: 0.88–1.40) (Table S1). In this group, mortality rates increased as age increased and were over four‐fold higher among 20+ years olds (4.46/100 person‐years; 95% CI: 3.09–6.21) compared to 15–19 years (0.98/100 person‐years; 95% CI: 0.68–1.37) and 10–14 years (0.32/100 person‐years; 95% CI 0.14–0.63) (Figure 1). Among adolescents and youth living with sexually acquired HIV, the overall mortality incidence rate was 1.05/100 person‐years (95% CI: 0.66–1.58), similar to the overall mortality incidence rate estimated among those living with vertically acquired HIV. In this group, there were no significant differences in mortality rates across age groups or by sex.

**Figure 2:**
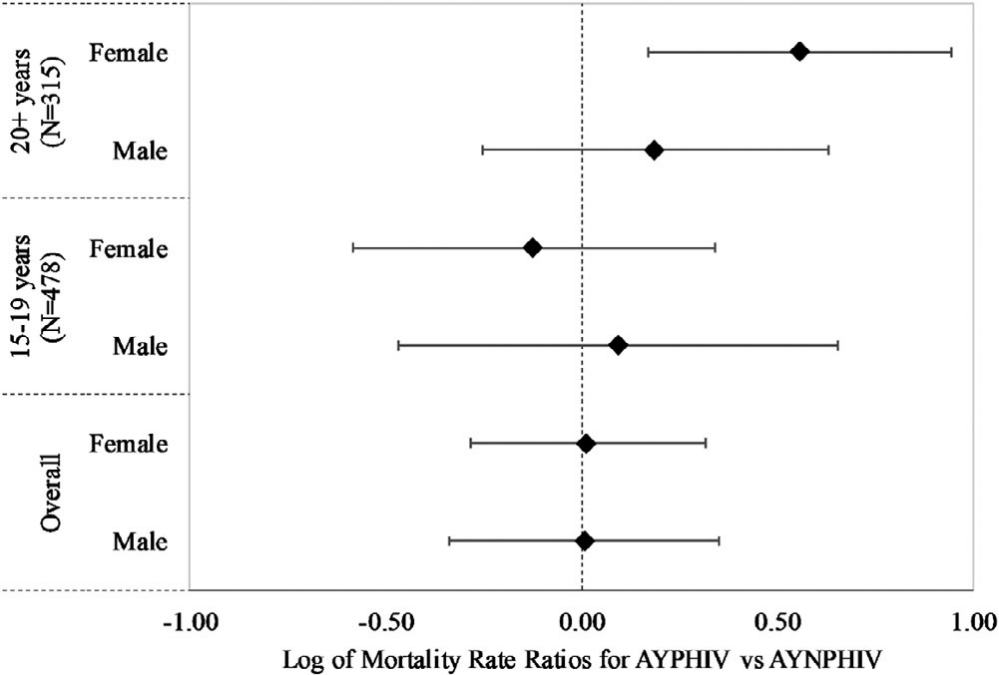
Mortality incidence rate ratios (IRRs) comparing mode of HIV acquisition groups, stratified by sex and time‐varying age. *The 10–14 age group was dropped from Figure 2 as there were no deaths in the sexually acquired HIV group, such that the ratio is undefined. AYNPHIV, adolescents and youth living with non‐perinatally acquired HIV; AYPHIV, adolescents and youth living with perinatally acquired HIV.

Figure 2 and Table S2 show the mortality IRRs comparing vertical versus sexual acquisition groups, stratified by sex and time‐updated age, adjusted for time on ART. Overall, we found no significant differences in mortality incidence rates between these HIV acquisition groups, in both males and females. These results were similar for the 15‐ to 19‐year‐olds. However, for 20+ year‐olds, we found a higher risk of mortality for vertical compared to the sexual HIV acquisition group among females (IRR: 3.61, 95% CI: 1.48–8.82).

Overall, age, sex and mode of HIV acquisition were significantly associated with mortality among ALHIV (Table 3). Older age groups: 15–19 year‐olds (IRR: 3.77, 95% CI 1.74–8.16) and 20+‐year‐olds (IRR: 9.98, 95% CI 4.59–21.7) were associated with a higher risk of mortality relative to 10‐ to 14‐year‐olds, males (IRR: 1.78, 95% CI 1.16–2.72) were associated with 1.78 times higher mortality relative to females, while adolescents living with vertically acquired HIV (IRR: 2.03, 95% CI 1.12–3.65) were associated with 2.03 times higher mortality than those living with sexually acquired HIV.

**Table 3:** Multivariable Poisson regression model for overall adjusted mortality incidence rate ratios among ALHIV (*N* = 1107)

Factors	IRRs (95% CI)	*p*‐value
Age group (time‐updated)		
10–14 years (reference)	1	
15–19 years	3.77 (1.74–8.16)	0.001
20+ years	9.98 (4.59–21.7)	<0.001
Baseline factors		
Male	1.78 (1.16–2.72)	0.009
Adolescents and youth living with vertically acquired HIV	2.03 (1.12–3.65)	0.019
Rural residence	0.79 (0.47–1.32)	0.366
Time on ART (years)	0.96 (0.91–1.02)	0.144
Food insecurity	1.24 (0.74–2.08)	0.407
Lack of access to basic necessities	1.22 (0.76–1.95)	0.406
Double orphanhood	0.99 (0.58–1.69)	0.974
Not enrolled in school	1.18 (0.51–2.74)	0.707

Abbreviation: 95% CI, 95 percent confidence interval.

### 3.2 All‐cause mortality rates by ART adherence

**Figure 3:**
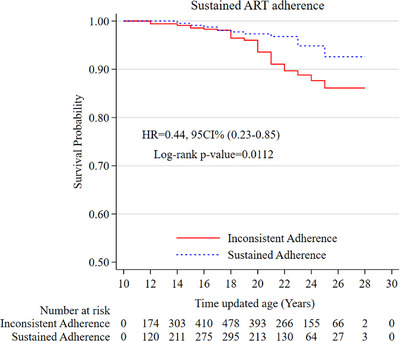
Kaplan–Meier estimates of all‐cause mortality by antiretroviral treatment (ART) adherence (*N* = 933). CI, confidence interval; HR, hazard ratio for adolescents.

A total of 933 ALHIV were interviewed across all three time points and were categorized into four longitudinal ART adherence trajectories [23]. The proportion of participants who died after the third follow‐up by adherence trajectory group was: 8.2% in the gradually decreasing, 5.3% in the low start and increasing, 5.1% in the consistent adherence and 9.1% in the low start and decreasing group. In total, 6.1% (*N* = 57) of adolescents and youth died after the third follow‐up, with noticeably different risks of mortality by trajectory membership (Figure S1). The results exhibit a dose‐dependent relationship—although not statistically significant—between mortality and various adherence trajectories, with mortality decreasing systematically as ART adherence improves. Overall, compared to all other trajectory groups combined, adolescents with sustained ART adherence (consistent adherence trajectory) had significantly higher survival rates (log‐rank *p* = 0.0112) (Figure 3). The risk of death was 55% lower in adolescents with sustained adherence than in those with inconsistent adherence over time (aHR: 0.45, 95% CI 0.23–0.88; *p* = 0.020) after adjusting for covariates (Table S3). A sensitivity analysis using the mid‐point date between the date of last contact and the median follow‐up date for all participants as the date of death showed consistent findings (Table S4).

## 4 DISCUSSION

This study provides disaggregated data on all‐cause mortality from a large prospective cohort of adolescents in South Africa. We observed substantially higher mortality rates among ALHIV compared to HIV‐negative peers, with findings suggesting that both HIV‐positive status and socio‐economic vulnerabilities contribute to a higher risk of death among ALHIV. We further observed significantly higher mortality rates among ALHIV males compared to ALHIV females. Overall, our results showed that mortality increases with age, with males and adolescents and youth living with vertically acquired HIV at a higher risk of mortality. When comparing adolescents’ mode of HIV acquisition, stratified by age groups and sex, females aged 20+ years with vertically acquired HIV had a higher risk of mortality compared to those with sexually acquired HIV of the same age group. Furthermore, adolescents with sustained adherence had significantly lower rates of all‐cause mortality (aHR: 0.42; 95% CI 0.20–0.87).

Despite notable declines in mortality among people living with HIV since the introduction of ART [28], we observed significantly higher all‐cause mortality among ALHIV compared to HIV‐negative peers, similar to those reported in other studies [29, 30, 31]. Although survival has improved considerably among ALHIV due to advancements in ART and care, a significant gap remains in this population compared with the general population. We further found marked sex differences in the proportions of deaths, with higher mortality among males than in females. These sex differences have been noted in several studies [15, 30, 32]. For example, a Global Burden of Disease Study assessing the global burden of adolescent mortality showed that sex differences in adolescent mortality continue to widen globally [15]. In South Africa, the increased mortality risk in men aged 20+ years is consistent with national trends, where young men experience a disproportionate burden of deaths from violence, road traffic accidents, injuries and substance misuse [33, 34]. Additionally, poor male engagement in care [35] may contribute to sex disparities [36]. As adolescents age into adulthood, more data on the cause of death will be needed to allow for more accurate assessments.

Our study also found that adolescents with vertically acquired HIV have twice the risk of mortality than those with sexually acquired HIV; these findings are similar to those reported in other cohorts of ALHIV in Southern Africa [32, 37]. For older adolescents and youth aged 20+ years, we observed significantly higher mortality rates (3.61 times) among those with vertically acquired HIV compared to those with sexually acquired HIV. Female adolescents who have acquired HIV more recently may be benefiting from early diagnosis, improved care and modern ART availability. In turn, this group may have a lower risk of mortality compared to female adolescents with vertically acquired HIV [4], a group with long‐term exposure to limited paediatric formulations of ART regimens [38]. Moreover, as female adolescents living with vertically acquired HIV transition to adulthood, they become increasingly responsible for their health and move away from caregiver‐supported care, which may impact their treatment outcomes [39]. Lastly, older female adolescents and youth aged 20+ years are of reproductive age, and may experience challenges related to pregnancy, with those living with vertically acquired HIV at greater risk of pregnancy‐related complications [40, 41].

This analysis also found that ALHIV with sustained ART adherence had a 58% lower risk of death compared to those who had inconsistent adherence over the study follow‐up period. The observed dose‐dependent relationship between mortality and various adherence trajectories underscores the importance of sustaining high adherence to reduce mortality risk. These findings are consistent with research documented in other low‐ and middle‐income countries [42, 43, 44] that demonstrate that adherence to ART can prolong survival among ALHIV. Empirical research suggests that poor ART adherence leads to viral non‐suppression, reductions in CD4 count, increased HIV transmission risk and subsequent clinical failure, which increases the risk of death [45]. Evidence from global cohorts also shows that ART interruptions, which can lead to poor adherence, are associated with increased mortality [46, 47]. Therefore, sustained adherence to ART is critical to the survival of ALHIV.

This study has several limitations. First, it describes all‐cause mortality, as we were unable to ascertain the cause of death for participants. Further studies could investigate the cause of death among adolescents, which was not possible with this dataset. Second, almost one‐tenth of adolescents were lost to study follow‐up or had families unable to be contacted, leading to gaps in the verification of mortality. Third, the use of median dates for the missing date of death may bias the estimated rates of mortality. Fourth, the focus on adolescents 10–19 years results in a survivor bias, as individuals with vertically acquired HIV would have had to survive childhood to be eligible for inclusion. Fifth, while the mode of HIV acquisition was determined using standard methods and validated using a detailed algorithm that considered other factors, some misclassification may still occur due to inherent limitations in self‐reported data. We also acknowledge that the higher LFTU among males likely led to an underestimation of mortality rates in this group. The key strength of this study is that we used appropriate HIV‐negative peers as a comparison group.

## 5 CONCLUSIONS

This study provides mortality data disaggregated by HIV status, age and sex, as well as comparing mortality by mode of HIV acquisition. Consistent with previous studies in sub‐Saharan Africa, our findings indicate that ALHIV experience significantly higher mortality rates than their HIV‐negative peers, despite substantial improvements in HIV care and treatment. This persistent gap in mortality highlights the ongoing vulnerabilities faced by ALHIV, including barriers to ART adherence, limited access to adolescent‐friendly healthcare and broader structural inequalities in resource‐constrained settings. Our findings also reinforce the critical role of interventions to reduce mother‐to‐child HIV transmission and associated mortality risks, aligning with global efforts to eliminate perinatal HIV transmission. Furthermore, there is a need to improve the availability and quality of mortality data, including cause‐of‐death reporting in ALHIV, to inform UNAIDS estimates and guide policy in low‐resource settings [14]. Future research should explore the specific social and structural determinants contributing to mortality disparities among ALHIV to design more effective interventions.

## COMPETING INTERESTS

No competing risks to declare.

## AUTHORS’ CONTRIBUTIONS

SZ conceptualized and led the statistical analyses, including writing the full draft of the manuscript. ET, BG, JK, LFJ, JT, WS, LK and LC reviewed and provided feedback on the manuscript content. All authors approved the final draft. ET and LC designed and implemented the overall study.

## FUNDING

This project was made possible partly by a CIPHER grant from the International AIDS Society [155‐Hod; 2018/625‐TOS]; Claude Leon Foundation [F08 559/C]; the South African National Department of Social Development [27/2011/11 HIV AND AIDS]; Evidence for HIV Prevention in Southern Africa (EHPSA); a UK aid programme managed by Mott MacDonald; the University of Oxford's ESRC Impact Acceleration Account [K1311‐KEA‐004]; Janssen Pharmaceutica N.V., part of the Janssen Pharmaceutical Companies of Johnson & Johnson; jointly funded by the UK Medical Research Council (MRC) and the Foreign Commonwealth and Development Office (FCDO) under the MRC/FCDO Concordat agreement, together with the Department of Health and Social Care (DHSC); the Nuffield Foundation; the Oak Foundation [OFIL‐20‐057]; Oxford University Clarendon‐Green Templeton College Scholarship; the Regional Inter‐Agency Task Team for Children Affected by AIDS—Eastern and Southern Africa (RIATT‐ESA); the Philip Leverhulme Trust [PLP‐2014‐095]; UNFPA South Africa; UNICEF Eastern and Southern Africa Office (UNICEF‐ESARO); the John Fell Fund [161/033]; the European Research Council (ERC) under the European Union's Horizon 2020 research and innovation programme (n° 771468); the UKRI GCRF Accelerating Achievement for Africa's Adolescents (Accelerate) Hub (Grant Ref: ES/S008101/1); the Fogarty International Center, National Institute on Mental Health, National Institutes of Health under Award Number (K43TW011434 and D43TW011308); University of Cape Town (UCT) Vice Chancellor 2030 Future Leaders programme. This research is also partly supported by the National Research Foundation (NRF) of South Africa (Grant Number: 138070).

## DISCLAIMER

The views expressed in written materials or publications are solely the responsibility of the authors and do not represent the official views of the National Institutes of Health, the Nuffield Foundation or the official policies of the International AIDS Society.

## Supporting information




**Table S1**: Mortality incidence rates (per 100 person‐years) and 95% confidence interval among adolescents and youth living with HIV and on antiretroviral treatment during follow‐up, by sex and time‐updated age.
**Table S2**: Mortality incidence rate ratios (IRRs) among adolescents and youth living with vertically acquired HIV compared with those living with sexually acquired HIV, stratified by sex and time‐updated age.
**Figure S1**: Kaplan‐Meier estimates of the cumulative incidence of all‐cause mortality by trajectory group (N = 933).
**Table S3**: Adjusted Cox proportional hazards model results: Predicting all‐cause mortality by sustained antiretroviral treatment (ART) adherence (N = 933)
**Table S4**: Predicting all‐cause mortality by sustained antiretroviral treatment (ART) adherence: A sensitivity analysis using an alternative date of death definition (N = 933).

## Data Availability

The data that support the findings of this study are available for non‐profit use upon reasonable request following study data sharing protocols available here.
