# Supplementary material for: Age‐specific all‐cause mortality rates among adolescents and youth living with and without HIV: Evidence from a cohort study in South Africa
Source: J Int AIDS Soc. 2025 Jun 25;28(6):e26522. doi: 10.1002/jia2.26522 (PMC12188098; doi:10.1002/jia2.26522)
Supplement: Supplementary file 1 — Table S1: Mortality incidence rates (per 100 person‐years) and 95% confidence interval among adolescents and youth living with HIV and on antiretroviral treatment during follow‐up, by sex and time‐updated age. Table S2: Mortality incidence rate ratios (IRRs) among adolescents and youth living with vertically acquired HIV compared with those living with sexually acquired HIV, stratified by sex and time‐updated age. Figure S1: Kaplan‐Meier estimates of the cumulative incidence of all‐cause mortality by trajectory group (N = 933). Table S3: Adjusted Cox proportional hazards model results: Predicting all‐cause mortality by sustained antiretroviral treatment (ART) adherence (N = 933) Table S4: Predicting all‐cause mortality by sustained antiretroviral treatment (ART) adherence: A sensitivity analysis using an alternative date of death definition (N = 933). [file JIA2-28-e26522-s001.docx]

**Supporting Tables and Figures**

**Table S1: Mortality incidence rates (per 100 person-years) and 95% confidence interval among adolescents and youth living with HIV and on antiretroviral treatment during follow-up, by sex and time-updated age.**

|  | **Adolescents and youth living with perinatally acquired HIV** | | | **Adolescents and youth living with non-perinatally acquired HIV** | | |
| --- | --- | --- | --- | --- | --- | --- |
| **Time-updated age** | **Male** | **Female** | **Overall** | **Male** | **Female** | **Overall** |
| 10-14 years | 0.45  (0.17-0.98) | 0.17  (0.02-0.61) | **0.32**  **(0.14-0.63)** | - | - | - |
| 15-19 years | 1.18  (0.71-1.84) | 0.79  (0.43-1.33) | **0.98**  **(0.68-1.37)** | 0.91  (0.19-2.64) | 0.76  (0.25-1.77) | **0.81**  **(0.35-1.60)** |
| 20+ years | 5.66  (3.39-8.80) | 3.56  (2.01-5.81) | **4.46**  **(3.09-6.21)** | 3.18  (1.04- 7.28) | 1.02  (0.47- 1.93) | **1.35**  **(0.74-2.25)** |
| **Overall** | **1.32**  **(0.96-1.78)** | **0.92**  **(0.62-1.30)** | **1.12**  **(0.88-1.40)** | **1.52**  **(0.66-2.97)** | **0.89**  **(0.49-1.49)** | **1.05**  **(0.66-1.58)** |

**Table S2: Mortality incidence rate ratios (IRRs) among adolescents and youth living with vertically acquired HIV compared with those living with sexually acquired HIV, stratified by sex and time-updated age.**

|  | **Male** | | **Female** | |
| --- | --- | --- | --- | --- |
|  | **IRR** | **95% CI^b^** | **IRR** | **95% CI^b^** |
| **Overall** | **1.02** | **(0.46-2.25)** | **1.03** | **(0.52-2.07)** |
| **Age-group** |  |  |  |  |
| 10-14 years | - | - | - | - |
| 15-19 years | 1.24 | (0.34-4.52) | 0.75 | (0.26-2.19) |
| 20+ years | 1.54 | (0.56-4.29) | 3.61 | (1.48-8.82) |

.

**Figure S1: Kaplan–Meier estimates of the cumulative incidence of all-cause mortality by trajectory group (N*=* 933).**


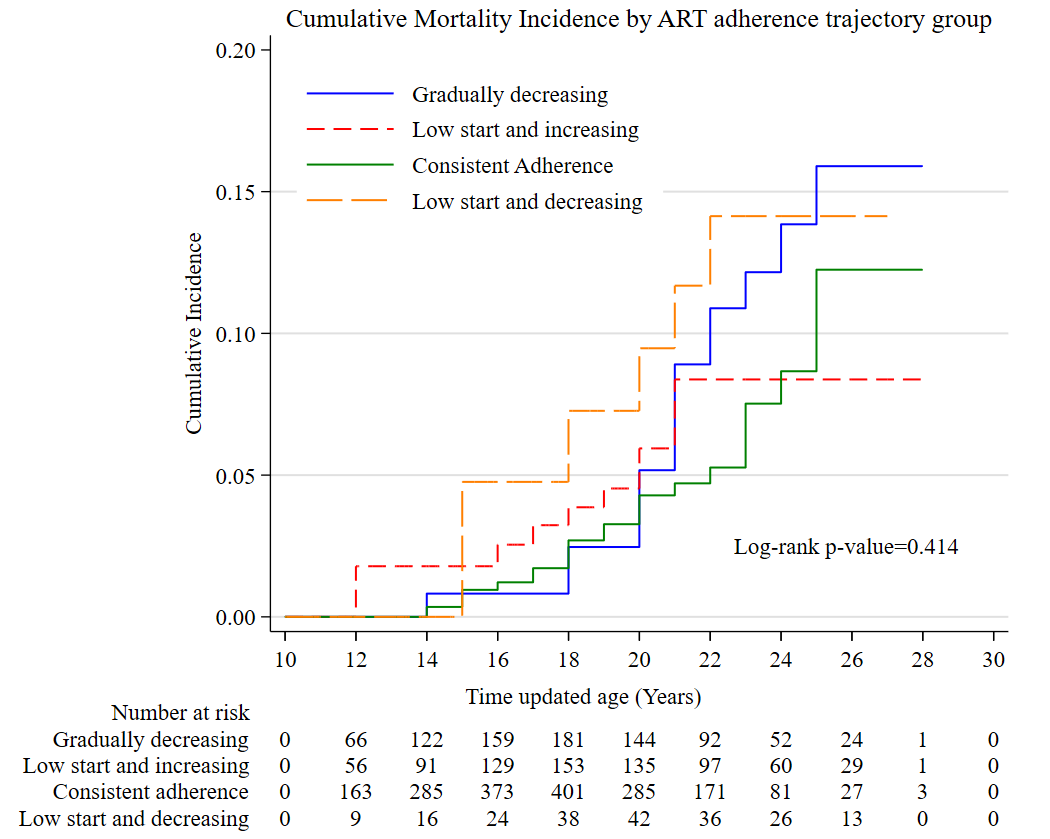


**Table S3: Adjusted Cox proportional hazards model results: Predicting all-cause mortality by sustained antiretroviral treatment (ART) adherence (N=933)**

| **Factors** | **aHR (95%CI)** | **P-value** |
| --- | --- | --- |
| Sustained adherence | 0.45 (0.23 - 0.88) | **0.020** |
| **Baseline controls** |  |  |
| Age (years) | 1.03 (0.92-1.15) | 0.569 |
| Male | 1.86 (1.05-3.28) | **0.033** |
| Rural residence | 0.69 (0.35-1.38) | 0.300 |
| Time of ART (years) | 0.97 (0.90-1.05) | 0.527 |
| Adolescents and youth living with vertically acquired HIV | 1.43 (0.66-3.10) | 0.357 |
| Food insecurity | 1.75 (0.93-3.29) | 0.087 |
| Lack of access to basic necessities | 1.02 (0.54-1.92) | 0.945 |
| Double orphanhood | 1.08 (0.53-2.20) | 0.827 |
| School enrolment | 1.01 (0.31-3.37) | 0.981 |

**Table S4: Predicting all-cause mortality by sustained antiretroviral treatment (ART) adherence: A sensitivity analysis using an alternative date of death definition (N=933).**

| **Factors** | **aHR (95%CI)** | **P-value** |
| --- | --- | --- |
| Sustained adherence | 0.45 (0.23 - 0.89) | **0.021** |
| **Baseline controls** |  |  |
| Age (years) | 1.03 (0.94-1.28) | 0.366 |
| Male | 1.68 (1.04-2.98) | **0.023** |
| Rural residence | 0.67 (0.34-1.36) | 0.271 |
| Time of ART (years) | 0.98 (0.91-1.06) | 0.611 |
| Adolescents and youth living with vertically acquired HIV | 1.49 (0.68-3.25) | 0.318 |
| Food insecurity | 1.96 (0.97-3.67) | 0.068 |
| Lack of access to basic necessities | 1.05 (0.56-1.96) | 0.883 |
| Double orphanhood | 1.10 (0.54-2.24) | 0.788 |
| School enrolment | 1.16 (0.36-3.77) | 0.796 |
